# Advances in the Development and the Applications of Nonviral, Episomal Vectors for Gene Therapy

**DOI:** 10.1089/hum.2020.310

**Published:** 2021-10-18

**Authors:** Grace Elizabeth Mulia, Virginia Picanço-Castro, Eleana F. Stavrou, Aglaia Athanassiadou, Marxa Leão Figueiredo

**Affiliations:** ^1^ Department of Basic Medical Sciences, Purdue University College of Veterinary Medicine, West Lafayette, Indiana, USA.; ^2^ Center for Cell-based Therapy (CTC), Regional Blood Center of Ribeirão Preto, University of São Paulo, Ribeirão Preto, São Paulo, Brazil.; ^3^ Department of General Biology, Medical School, University of Patras, Patras, Greece.

**Keywords:** gene delivery, plasmid, nonviral, episome, gene therapy, S/MAR

## Abstract

Nonviral and nonintegrating episomal vectors are reemerging as a valid, alternative technology to integrating viral vectors for gene therapy, due to their more favorable safety profile, significantly lower risk for insertional mutagenesis, and a lesser potential for innate immune reactions, in addition to their low production cost. Over the past few years, attempts have been made to generate highly functional nonviral vectors that display long-term maintenance within cells and promote more sustained gene expression relative to conventional plasmids. Extensive research into the parameters that stabilize the episomal DNA within dividing and nondividing cells has shed light into the genetic and epigenetic mechanisms that govern replication and transcription of episomal DNA within a mammalian nucleus in long-term cell culture. Episomal vectors based on scaffold/matrix attachment regions (S/MARs) do not integrate into the genomic DNA and address the serious problem of plasmid loss during mitosis by providing mitotic stability to established plasmids, which results in long-term transfection and transgene expression. The inclusion, in such vectors, of an origin of replication—initiation region—from the human genome has greatly enhanced their performance in primary cell culture. A number of vectors that function as episomes have arisen, which are either devoid or depleted of harmful CpG sequences and bacterial genes, and their effectiveness, as well as that of nonintegrating viral episomes, is enhanced when combined with S/MAR elements. As a result of these advances, an “S/MAR technology” has emerged for the production of efficient episomal vectors. Significant research continues in this field and innovations, in combination with promising systems based on nanoparticles and potentially combined with physical delivery methods, will enable the generation of optimized systems with scale-up and clinical application suitability utilizing episomal vectors.

## Introduction

The gene therapy sector has experienced accelerated growth in the past few years and although viral-based vectors are still used in most clinical trials, because of their high effectiveness, episomal vectors have been reemerging in the patent landscape, as shown by a recent report from our group.^1^ Episomal vectors or “episomes” are free, circular, extrachromosomal DNA molecules of viral and nonviral origin. Viral episomes refer to viruses whose natural life cycle includes a stage of remaining as free, viral DNA within the cell nucleus, retaining the ability to encode proteins without integrating into the host cell's genome.^2^ Examples include Adenoviruses, a family of DNA viruses, or the adeno-associated virus (AAV), a small, nonpathogenic satellite virus. Nonviral episomes are effectively plasmids, usually of a larger size relative to conventional plasmids, and can exist independent of the genomic DNA for longer periods than conventional plasmids. Both types of episomes are used as vectors of gene transfer in gene therapy applications and have undergone extensive modifications for achieving higher safety as well as higher efficiency in basic parameters such as transfection, establishment, and high-level transgene expression. An in-between case is that of nonintegrating viruses, which have been derived from integrating viruses engineered to be integrase deficient, for example, nonintegrating lentiviruses.^3,4^ Nonintegrating viral vectors present an interesting development to the field and can provide stable transgene expression in quiescent as well as proliferating cells, as it has been reviewed recently.^5^ These vectors may be reinforced with scaffold/matrix attachment regions (S/MARs), yet they still harbor viral sequences in their genome and thus can induce an immune response. In addition, they have a limited packing capacity and high production cost.^3,6^

Another type of nonviral gene transfer system is the Sleeping Beauty (SB) transposon system for gene transfer, which is a category in itself and has emerged as a promising alternative to viral vectors, presented recently in a comprehensive review.^7^ The SB system shares with the viral vectors the need to integrate into the recipient cell's genome to be functional. However, contrary to viral vectors, it does not show integration preference for sites within highly transcribed genome domains. And since the vast majority of the genome does not contain highly transcribed genome domains, random integration of SB outside these domains reduces its risk of insertional mutagenesis.^7^ Nevertheless, considering that a great deal of the genome is transcribed to some extent, more characterization is likely necessary to determine the full safety profile of the SB system.

Owing to safety as a major advantage relative to the vectors described in the preceding paragraphs, in this review, we focus on nonviral, episomal vectors. In fact, episomal, nonintegrating vectors are emerging as a valid, alternative technology to integrating viral vectors for gene therapy, based on a lower risk for insertional mutagenesis, as well as a lesser potential for innate immune reactions. This article provides an overview of the advances in the main categories of episomal vectors, including an array of nonviral episomal vectors and the investigations that provided fundamental knowledge on their nature and function. The goal of this review is to highlight improvements made to these episomal vectors and outline specific changes in their backbones that allowed their optimization and expansion into preclinical studies. We envisage that episomes are well suited as the next generation of vectors to be explored in emerging therapeutic and clinical trials.

## The Nonviral Episomal Vectors: Advantages and Challenges

Viral systems^1^ remain the most efficient gene delivery tools, however, they also have several limitations and drawbacks—the most important being the risk of insertional mutagenesis, resulting from vector integration into the cellular genomic DNA. Compared to viral systems, nonviral vectors present four main advantages, since they (i) generate fewer concerns regarding biosafety and can be delivered repeatedly, (ii) harbor a greater transgene capacity (relative to some viruses), (iii) are easier to characterize chemically and produce on a large scale with significantly less cost and high reproducibility, and (iv) rarely integrate.

**Figure 1.**
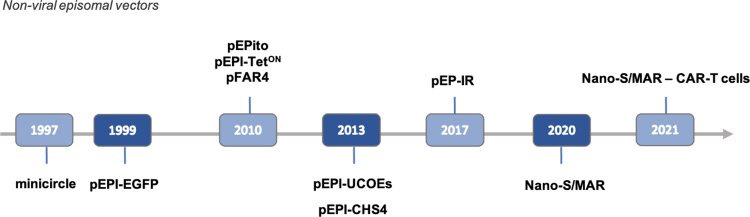
A timeline showing selected key milestones in nonviral episomal gene therapy development. The respective vector descriptions with references are provided in the text and in Figs. 2–5. Color images are available online.

Nevertheless, inherent to nonviral vectors are also important challenges. First, nonviral systems face appreciable plasmid loss during their mitotic segregation in dividing cells. Second, silencing of transgene expression may occur due to epigenetic events. Third, there is variable efficiency of plasmid delivery into various cells, both by physical and chemical methods. To overcome these challenges, improvements have been explored extensively by various groups to further enhance and secure the stability and potency of episomes *in vitro* and *in vivo*. These may include strategies aimed at enhancing transfection efficiency, vector establishment, and vector maintenance in the host cell nucleus, as well as decreasing plasmid loss or cell toxicity. The combination of some or several of these strategies could prove transformative in facilitating nonviral episomal DNA vector translation as increasingly favorable gene therapy tools. Figure 1 shows a timeline of some key milestones in episomal-related gene therapy developments.

### The gene delivery systems for nonviral, episomal vectors

Successful episome-based gene expression hinges on the ability to safely and precisely deliver these episome vectors to complex biological environments. Although bare nucleic acids can be delivered *in vivo* by direct introduction of DNA or RNA into cells, rapid clearance and loss of expression limit the effectiveness of this approach.^8^ Several carrier systems can be utilized to deliver episomes, and these include liposomes, synthetic polymers, or physical means of gene delivery (electroporation and sonoporation, among others). Liposomes and synthetic polymers are chemical means that exploit the nanoscale size and controllable surface properties of organic and polymeric molecules for gene delivery. These chemical carriers are intended to match or exceed the performance of viral vectors with fewer immunogenic complications. In addition, virus-like particles represent another class of nonviral gene delivery vectors, and these typically consist of self-assembled viral protein nanostructures.

Physical delivery methods destabilize membranes as a mechanism for introducing genetic material. Importantly, these gene delivery methods need not be used in isolation and there are many examples of combining different methods, especially chemical and physical, to enhance gene delivery *in vitro* and *in vivo*. Electroporation is a means of physical gene transfer, first described in the 1980s,^9^ which utilizes a pulsed electric field to introduce DNA into cells, exploiting the weak interactions of lipid bilayers to create membrane pores. Electroporation has been applied extensively in gene transfer, DNA vaccination, and drug delivery, and is the mode of delivery for most plasmids used in recent and ongoing clinical trials.^10^ Major ongoing challenges in electrogene transfer include variable transfection efficiency in different tissues and a lack of targeting. Electroporation also tends to promote high cell toxicity, with extensive cell loss, especially in primary cells, which are in limited supply per patient.^11^ In another physical approach, pores are generated through the use of ultrasound (sonoporation), typically in the presence of microbubbles, exploiting the weak interactions of lipid bilayers to promote acoustic streaming of genetic material into cells.^12^ Sonoporation also can facilitate local transport across more complex biological structures, such as skeletal muscle,^13^ vasculature, and solid tumors.^14^ Despite the many advantages of physical approaches described, major unsolved challenges include variable efficiency levels in different tissues, and, by default, a lack of targeting.

Polymers, liposomes, or other nanoscale structures can be modified to enhance their biocompatibility, reduce cytotoxicity (*e.g.,* polyethylene glycol [PEG]), and augment their potential to target specific tissues or cell types. Polymer and liposome chemists increasingly collaborate with biologists to optimize the capability of synthetic nanostructures for improving nonviral gene delivery. Significant advances emerging from these partnerships include novel polymer designs for polyplex formation, targeted systems with enhanced efficacy, and advanced synthetic constructs with improved stability when interacting with proteins and erythrocytes in blood.^15^ More recently, novel PEGylated siRNA-loaded lipid nanoparticles have addressed the greatest challenge in implementing siRNA therapeutics, which is their delivery.^16^ Episomes have so far been successfully delivered in several tissues using lysine-PEG^17^ and polycationic comb polymers with nuclear localizing sequences,^18^ among others. Continued efforts to target complexes or improve the transfection efficiency in tissues of interest will be important to realize the full therapeutic potential of episomes.

## The Development of Nonviral, Episomal Vectors

It is generally accepted that the major functions of the DNA in higher eukaryotes, namely the regulation of replication and transcription, are taking place within the three-dimensional nuclear matrix structures upon which chromosomal domains of higher chromatin order reside, in coordination with epigenetic factors, so that the particular program of genome structure gene expression is dynamic in each cell.^19^ The development of nonviral episomal vectors requires the identification of features residing either in the nucleus or in the plasmid and the elucidation of their interactions that affect the fate of the plasmid within the recipient cell.

### Genomic features that enhance the efficiency of nonviral, episomal vectors

**Figure 2.**
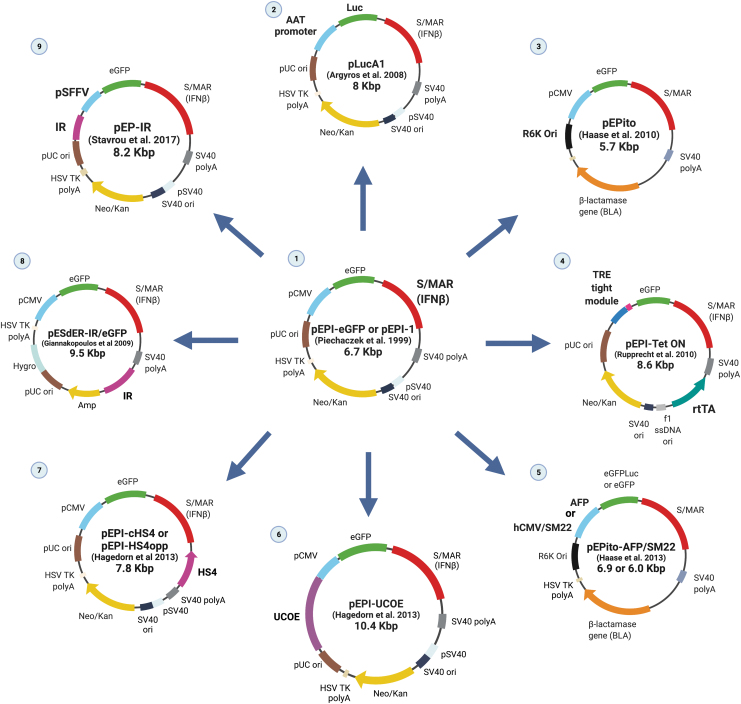
A family of pEPI-derivative plasmids, with examples of several modifications made to enhance each specific vector function. (1) The original vector pEPI (pEPI-eGFP or pEPI-1) was based on a pGFP-C1 commercial plasmid modified by insertion of a human β-interferon gene (IFNβ) S/MAR element,^21^ to promote episome retention in the host nucleus. Overtime, the family of pEPI derivatives grew through the addition or deletion of various genetic elements. (2) The pLucA1, containing a liver-specific promoter and a Luc transgene. (3) The pEPito, constructed by cloning the pEPI-1 plasmid replicon into the *Escherichia coli* plasmid R6K gamma origin, a plasmid backbone depleted of CpG motifs, and by omitting a second transcription unit, showed a more persistent transgene expression profile *in vivo* relative to the pEPI-1 replicon.^23^ (4) The pEPI-Tet ON combined the TRE fused to a minimal CMV promoter from the plasmid pTRE-Tight and the rtTA fused to a CMV promoter.^24^ pEPI-Tet ON is inducible by doxycycline in the presence of rtTA, which is able to bind to TRE and induce transcription of the transgene (eGFP). (5) The pEPito-AFP/SM22 vectors, with either a tumor-specific AFP promoter or the muscle-specific SM22 promoter,^100^ supporting transgene expression specifically in AFP^+^ human hepatocellular carcinoma (HUH7) or SM22^+^ cell lines, respectively. (6) The pEPI-UCOE, the UCOE-mediated enhanced transgene expression^25^ relative to any other genetic element examined.^104^ (7) In the pEPI-cHS4, the cHS4 insulator sequence increased vector establishment efficiency,^25^ and was shown to be the main element directing the vector to the preferred sites of establishment.^43^ (8) The pESdER-IR/eGFP carries the backbone of the pCEP4 plasmid. In this vector, the IR for replication was added to the pES/eGFP plasmid carrying the transcription cassette CMV-eGFP-S/MAR to restore plasmid replication in Jurkat cells, which was stalled following addition of the S/MAR element. This is the only case known of an S/MAR-containing vector having a detrimental effect.^26^ (9) The pEP-IR had the promoter CMV replaced by SFFV and the addition of the IR element, a *bona fide* mammalian replicator, for efficient transfection of CD34^+^ hematopoietic progenitor cells.^27^ AFP, alpha fetoprotein; cHS4, chicken hypersensitive site 4; eGFP, enhanced green fluorescent protein; HSV, herpes simplex virus; IR, initiation region; Luc, luciferase; Neo/Kan, neomycin/kanamycin; ori, origin of replication; pCMV, human cytomegalovirus promoter; Puro, puromycin; rtTA, reverse transactivator protein; SFFV, spleen focus-forming virus; S/MAR, scaffold/matrix attachment region; SM22, smooth muscle 22; SV40, simian virus 40; TK, thymidine kinase; TRE, tetracycline response element; UCOE, ubiquitous chromatin opening element. Created with Biorender.com Color images are available online.

The nucleic architecture of the eukaryotic cell is characterized by the organization of distinct nuclear compartments, carrying chromosomal territories dedicated, either active or suppressive, to fundamental biological processes, principally to transcription and replication.^20^ Specific elements of the genome carry out permissive communication within each territory and such chromosomal elements have been used for plasmid modification, to render episomes efficient vehicles for gene transfer. Such genomic features have been included in episomal vectors, summarized in Fig. 2.

The most commonly used episomal vector has been the pEPI-1, which originated from pGFP-C1 with the addition of the *S/MARs anchoring elements*, which keep the chromosomal DNA tethered into the nuclear matrix or scaffold. The 2.1 kb AT-rich S/MAR is the most widely studied of chromosomal elements, derived from the 5′ end of the human β-interferon gene and is the one included in the prototype episomal vector known as pEPI-1-eGFP or pEPI-1 (Fig. 2).^21^ The pEPI-1 vector carries the simian virus 40 (SV40) origin (*ori*) for replication and the S/MAR element instead of a large T antigen gene, which normally acts upon the SV40 *ori* for autonomous plasmid replication. Thus, this vector tends to have a safer profile, as it does not code for any viral protein that usually evokes immunological reactions. In addition, pEPI-1 has been shown to exist in very few copies per cell since there is no *trans* acting protein to enhance replication. Moreover, it is the presence of the S/MAR element, rather than the absence of the large T antigen gene, that renders pEPI-1 an efficient episomal vector. This has led to a number of episomal vectors generated based on S/MAR technology, and studied in a variety of cells and tissues,^22^ which are described next and are included in Fig. 2.

Following the generation of pEPI-1, the vector pLucA1, containing a liver-specific promoter and a luciferase (Luc) transgene, was developed. Also generated was the pEPito, a vector depleted of CpG motifs and that shows a more persistent transgene expression relative to the pEPI-1,^23^ as well as inducible systems for controlling transgene expression (pEPI-Tet^ON^).^24^ Another advancement was the development of vectors containing *chromatin modulator elements* such as the *cHS4 insulator* from the chicken β-like globin gene cluster (pEPI-cHS4), or ubiquitous chromatin opening elements (UCOEs)^25^ (pEPI-UCOE). The latter vectors were efficient in promoting the formation of chromatin structures within the nuclear architecture of living cells. In another attempt to improve the episomal vector replication inside host cells, a *chromosomal origin of replication,* the human β-globin replicator, a *bona fide* mammalian replication initiation region (IR), was included to generate the vectors pEdER-IR and pEP-IR, which have been shown to rescue episomal replication,^26^ and promote replication and establishment in primary cells.^27,28^

### The fate of nonviral, episomal vectors within recipient cells

Most plasmids (natural or artificial) used in transfections, remain in the nucleus only transiently. Nonviral episomes have to pass through a number of critical stages, starting with gaining cellular entry, until they can modulate sustained, long-term maintenance within cells. The fate of pEPI-based, established, and nonintegrating episomal vectors is characterized by a sequence of fundamental stages inside the host cell: *establishment, replication, mitotic stability*, and *plasmid segregation* in daugther cells at mitosis. These are complex processes, mediated by specific chromatin structures and DNA features, and studies during the recent years have greatly expanded our understanding on how episomes can function as gene transfer vectors in a gene therapy context.

It has been established that plasmids, both replicating and nonreplicating, can recruit histones and assemble nucleosomes following transfer into a recipient cell and entry into the nucleus. This process is more organized in the case of replicating plasmids, which are essentially turning into *minichromosome* structures.^29^ Nevertheless, these replicating episomal vectors have often suffered from *transient expression*, either because they were prone to a gradual silencing of the transgene or due to mitotic instability, which refers to plasmid loss during successive mitotic divisions.^30^ As discussed briefly in the previous section, the first nonviral, episomal vector pEPI-1 included an S/MAR,^21^ which promoted *mitotic stability* leading to long-term nuclear retention of the episome and ensured proper *plasmid segregation* at mitosis, through interactions with nuclear matrix proteins. Studies with vector pEPI-1 also have shed light on the mechanism of vector's *establishment* in the host nucleus—a process by which the plasmid acquires the status of a replicon, which is crucial for reaching mitotic stability and long term persistence of the episome.^31^ Once established, these S/MAR-based plasmids undergo *replication* once per cell cycle.^21^

In the following sections, we will present how the most common chromosomal elements (*i.e.,* cHS4, UCOEs, S/MAR, and IR) in episomal vectors are affecting their establishment, replication, mitotic stability, and plasmid segregation and how these features render episomes as efficient gene delivery systems.

### The pEPI-based episomal vectors can block integration into the genome of recipient cells

The term *episome* was proposed by Francois Jacob and Elie Wollman in 1958 to describe extrachromosomal genetic material that may replicate autonomously or become integrated into the chromosome.^32^ Although the term *episome* is now somewhat interchangeable with the term *plasmid*, episomes are larger in size and are retained for a longer period of time following transfection of host cells. Episome integration is a rare event and can occur through canonical sequence-independent, nonhomologous end joining or microhomology-mediated end joining.^33,34^ Nevertheless, episomal integration must be inhibited if episomes are to become safe vehicles for gene therapy applications.

pEPI-1 was the first vector reported as nonintegrative in the long term following transfection and the maintenance of key vector regions, such as SV40 ori or S/MAR, was found to be essential to avoiding plasmid integration.^21^ pEPI-1 derivatives, with a permissive expression cassette preceding the S/MAR module (*i.e.,* transcriptionally active), replicate episomally in Chinese hamster ovary (CHO) cells in association with the nuclear matrix, as demonstrated both by fluorescence *in situ* hybridization (FISH) analyses and by *in vivo* cross-linking experiments.^35^ This system was used to elucidate the critical role of the S/MAR in preventing vector genomic integration. Specifically, it was documented that transcription starting at the upstream GFP gene has to include the S/MAR as CMV-GFP-S/MAR-poly A transcription cassette, in order for the vector to remain in a free episomal state.^36^ Deletion of the GFP gene or the insertion of a transcription termination poly A site between the GFP gene and the S/MAR resulted in integration of the vector.^36^ The lack of integration of pEPI-1 and its derivatives has been documented abundantly in cell lines^37^ as well as in primary cells.^27,28^ However, there was one case reported of the plasmid hβ-S/MAR integration in the genome of human K562 cells, following 3 months of culture, but not in the genome of murine erythroleukemia cell line (MEL) cells in the same study.^38^ This was attributed to chromosomal instability of the K562 chronic myeloid leukemia cells, which are trisomic for chromosome 11. However, since it appears that such chromosomal instability was not observed in other studies, including those using cosmid vectors of 38 kb^6^ and later studies using the pEPI-1-^37^ or pEPI-derived vectors,^27,28^ we propose that it is not a constant feature of K562 cells, but rather likely to be randomly connected to the K562 isolate used in that particular study.^38^ The exact epigenetic mechanism involved in pEPI-1 as a nonintegrating vector is not yet understood, but it seems that the full-length S/MAR is not required for completely preventing integration, as investigated by comparing between five smaller overlapping segments of S/MAR transfected into C2C12 muscle cells.^39^ In addition, the S/MAR from the murine c-myc gene^40^ could not prevent integration, suggesting this is not a property of all sequences identified as S/MAR.

### Establishment of nonviral, episomal vectors within the nucleus of recipient cells

Vector establishment in the host nucleus was always considered to be a stochastic process by which the plasmid becomes an autonomous replicon, but the full array of elements and events that mediate this transition only lately started to be unraveled. Establishment of the vector is a rare event, in the ballpark of ∼1–5%,^35^ and shown to occur in close contact with nuclear genomic transcription sites.^41^ Vector establishment was characterized extensively when the synthetic plasmid pEPI-1 was first presented as an episome highly promising for gene therapy applications,^21^ because of its long-term nuclear retention and lack of integration in the host cell's genome.

It was amply documented, by FISH analysis in combination with immunofluorescence techniques in interphase CHO nuclei, that the pEPI-1 episome co-localized with subnuclear structures that harbored chromosomal domains involved in gene expression and replication at the onset of S phase.^36^ However, to ensure the stable association with the active nuclear domain, an epigenetic programming must be in operation and of which the specific elements are still unknown.^36^ Evidently, the first condition for establishment of vector pEPI-1 is its landing on a functional nuclear compartment carrying active chromosomal domains for transcription (*e.g.,* transcription factories) and replication (*e.g.,* replication foci).^36^ It is noteworthy that similar data have also been reported for viral episomes.^42^

In studies that followed, genomic elements transferred in plasmids were analyzed for their capacity to guide the respective plasmids to places that are favorable for replication and transcription, in the recipient nucleus. Such elements were the chicken hypersensitive site 4 (cHS4) insulator and the UCOEs, which typically are involved in the formation of chromatin structure in the nucleus of mammalian cells.^25^ Within episomal vectors, these elements contributed to the formation of autonomously replicating episomes and had a positive effect on promoting higher levels of transgene expression from within the pEPI-1 vector. In addition, the cHS4 insulator also caused an increase in vector establishment from ∼12% for pEPI to ∼25% for pEPI-HS4, presumably through vector-nuclear matrix interactions, thus providing knowledge of a second element that can function to promote more efficient establishment of pEPI-1.^25^

Insights on episome establishment were provided by the work by Hagedorn *et al.*,^43^ through the determination of preferred, genomic sites of attachment in S/MAR-based replicons, for the first time in an autonomous replicon. The preferred attachment sites were obtained by mapping the actual contact sites of stably established replicons in HeLa cells with the powerful method of circular chromosome conformation capture (4C) technology.^43^

During establishment, pEPI-1-based replicons do not show chromosomal preference, but rather a replicon-specific contact pattern, residing preferentially within actively transcribed regions of the genome and close to potential origins of DNA replication, as documented in mixed cell populations or in individual clones. However, the replicon carrying the HS4 insulator within plasmid pEPI-HS4 displayed a distinct contact pattern that did not show dynamic behavior during mitosis, whereas the replicon carrying the β-globin intron 1 was found in a broad array of genomic sites. Importantly, the replicons' association with promoters and enhancers of a number of actively transcribed genes did not interfere with the gene expression program of the cell. Finally, although the replicon pEPI-HS4 preferentially associated with a subset of actively transcribed genes, no common characteristics of these loci were detected.^43^ Nevertheless, the knowledge of an insulator within an S*/*MAR-based replicon, capable of directing this replicon toward specific, preferred contact sites in the genome, brought a new dimension to the design of episomal vectors for guiding specific DNA sequences into specific nuclear compartments.

### The mitotic stability and maintenance of plasmid copy number in proliferating cells

**Figure 3.**
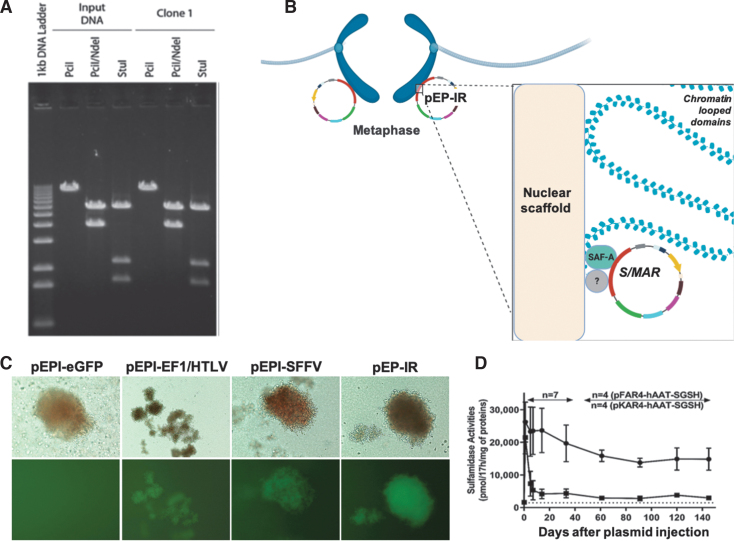
Mechanism of persistence of the pEP family of episome vectors in cells and tissues. **(A)** Persistence of episomes as detected by plasmid rescue assay. Restriction analyses, 4 months after transfection, of *pFAR4*–S/MAR-IN (input DNA) used for Huh7 cell transfections and of plasmid DNA (clone1) rescued from an *Escherichia coli* colony, transformed with HIRT extract for Huh7 cell transfections. Plasmid DNAs were digested with *Pci*I, *Stu*I, and *Pci*I/*Nde*I restriction enzymes and separated on agarose gel electrophoresis. Input DNA and clone 1 display the same restriction pattern denoting that the plasmids that exist for 4 months in the recipient cells are mitotic derivatives of the circular plasmids used to transfect the Huh7 cells. Slots are overloaded to ensure that a low abundance clone is not missed. Permissions to reprint obtained from reference^80^ publishers. **(B)** Mechanism of nuclear retention and mitotic stability of pEPI vectors, showing pEP-IR as a representative vector. These functions can be explained by S/MAR element interaction with metaphase chromosomes in a “piggy back”-like mechanism through binding the SAF-A protein (and possibly other proteins yet unknown?). Created withBioRender.com **(C)** Persistence of episomal vectors from CFC assays. Colonies of differentiated cells derived from CFC assays 14-day cultures (18 days after transfection) of FACS-sorted CD34^+^/eGFP^+^ cells from CD34^+^ cells transfected with episomal vectors, as indicated on top of the figure, all containing an S/MAR element: pEPI-eGFP control plasmid with CMV-eGFP; pEPI-EF-1/HTLV containing EF-1/HTLV-eGFP; pEPI-SFFV containing SFFV-eGFP; and pEP-IR containing IR and SFFV-eGFP. Phase contrast microscopy (*upper row*) and fluorescent microscopy (*lower row*) reveal that cell colonies expressing eGFP derive from the three experimental vectors; in contrast, cell colonies with the control plasmid pEPI-eGFP, carrying the promoter CMV that becomes nonfunctional in CD34^+^ derivative cells, there is lack of eGFP expression and hence of detectable fluorescence. Permissions to reprint obtained from reference^27^ publishers. **(D)** Sustained transgene expression over time when using episomes, showing pFAR4 as a representative vector. pFAR4 promotes sustained and elevated serum S-sulfamidase activity in liver. The plasmids pFAR4 and pKAR4 contain the eukaryotic expression cassette for murine sulfamidase gene—Sgsh cDNA, under the control of the liver-specific hAAT promoter, and the kanamycin resistance gene, respectively. Hydrodynamic injection of plasmids into the liver of WT mice resulted in sustained serum sulfamidase levels for plasmid pFAR4 for 140 days and at 11.3-fold than wild-type values (*dashed line*), while for pKAR4, there was a rapid decline of circulating sulfamidase protein during the same time frame. CFC, colony-forming cell; HTLV, human T cell leukemia virus long terminal repeat; SAF-A, scaffold attachment factor A. Permissions to reprint from D. Scherman and reference^79^ publishers. Color images are available online.

Mitotic stability of episomes is the process by which episomes become stably attached to the nuclear compartment site of establishment, while maintaining their autonomous replication. Lack of mitotic stability is manifested by plasmid loss during successive cell divisions, and this problem was successfully addressed by vector pEPI-1. The pEPI-1 vector achieved long-term episomal maintenance for 100 generations without selection as investigated by Southern blot and plasmid rescue analysis (Fig. 3A),^21^ and this has been repeatedly verified since.^27,28^

An aspect of the S/MAR element contribution to the formation of autonomous replicons refers to its specific interactions with the nuclear matrix, depicted in Fig. 3B. An abundant nuclear protein and constituent of the nuclear matrix or scaffold, SAF-A (scaffold attachment factor A) isolated from HeLa cell extracts, was shown in early studies to have high affinity for nuclear matrix/scaffold attachment DNA elements.^44^ Following the discovery of pEPI-1, the *in vivo* binding of the plasmid to SAF-A was documented in CHO cells using cross-linking and immunoprecipitation studies.^45^ Further studies with pEPI-1 confirmed that the vector is effectively maintained in active chromatin and is capable of association with most active nuclear domains.^36^ Importantly, FISH analysis of CHO cell metaphases transfected with pEPI-1 showed the plasmid's attachment to mitotic chromosomes at a high frequency and onto equivalent positions on sister chromatids,^36^ providing the first and crucial insight into the mechanism governing efficient plasmid segregation into daughter cells. The process has been described as similar to the “piggyback” mechanism utilized for Epstein-Barr virus (EBV) distribution in daughter cells, with the action of the EBV nuclear antigen (EBNA) protein, a virally coded transacting factor,^46^ and indicates that S/MARs might be involved in cohesion and separation of chromatids^47^ and therefore in the maintenance of the plasmid copy number of an autonomous replicon in proliferating cells.

### The autonomous replication of S/MAR-based plasmids and synergy with IR elements

The pEPI-1 vector replicates episomally in CHO cells and human cell lines at about 5 to 15 copy numbers and is mitotically stable in the absence of selection for several 100 generations,^21,37^ as well as in primary cells at a lower level for some of the larger episome plasmids (copy number of 1–3).^27,28^ It has been assumed that pEPI-1 uses the host's replication machinery and thus should co-replicate with the chromosomal DNA of the host cell. Evidence has showed that in stably transfected cell clones, pEPI-1 is associated with early replicating foci^36^ and replicates once per cell cycle, during early S phase.^31^ Replication of plasmid pEPI-1 as an autonomous, nonintegrating vector, thus seems to be under the influence of three characteristic features^36^: (i) the *SV40 ori* as an origin of replication, (ii) the presence of a transcriptionally active cassette upstream of the S/MAR element, and (iii) the presence of the S/MAR itself. It is understood that the process is mediated by epigenetic factors, but the tuning of these parameters in a temporal manner during vector replication is not yet known.

The role of the S/MAR element in pEPI-1-derived plasmids is multivalent and complex, depending on the vector and experimental context. Contrary to the initial findings from the first report on pEPI-1,^21^ a few plasmids devoid of the SV40 *ori* have been found to integrate, and the S/MAR has been found to be sufficient for replication and maintenance of episomes in mammalian cell*s* in the absence of the SV40 *ori.*^35^ In addition, the use of a tandem array of four 155 bp modules, designed to represent the core, unwinding S/MAR portion, constitutes the minimal sequence requirements for an S/MAR to function *in vivo*, and this is due presumably to the S/MAR sequence ability to recruit cellular replication factors.^35^ These data complement findings that the origin recognition complex (ORC) can bind onto the episomal DNA independent of the DNA sequence, and the initiation sites of DNA replication in episomes are determined epigenetically, as is the case with mammalian chromosomes.^31^ Shortened S/MAR regions were shown again to be sufficient for replication and maintenance of episomes in mammalian cells containing truncated S/MARs (positions 781–1,320, 1,201–1,740, and 1,621–2,201), with these regions retaining full activity and mediating episomal vector replication.^39^

The most dramatic effect ever exerted by the S/MAR element on the plasmid's replication was documented upon its transfer from the pEPI-1 plasmid to the commercially available pCEP4 plasmid, containing the EBV *ori*P and the EBNA1 gene.^26^ Following transfection of Jurkat cells with the pCEP4-S/MAR plasmid, replication was stalled and the culture died out within a week. Replication was rescued when the EBV *ori*P in plasmid pCEP4-S/MAR was replaced by the IR replicator, a *bona fide* mammalian IR of replication, that is, plasmid pESdER-IR/eGFP.^26^ The IR element derives from the human beta globin locus and it is genetically determined as a *Replicator*, in the sense that it can confer initiation of DNA replication in ectopic sites that lack such capacity.^48^ Further studies utilized the Stress Induced Duplex Destabilization analysis to document that, within vector pCEP4-S/MAR, the S/MAR created overwinding tension along the backbone sequences, precluding replication.^26^ This tension was released with inclusion of the IR (vector pCEP4-S/MAR-IR) exerting a strong chromatin opening activity, which is comparable only to the one exerted by the S/MAR element, while the remaining sites remain all in a closed chromatin configuration (plasmid pESdER-IR/eGFP).^26^

The IR was included in a pEPI-1-based episomal vector carrying the spleen focus-forming virus (SFFV) promoter, active in CD34^+^ cells, in place of the cytomegalovirus (CMV) promoter, driving enhanced green fluorescent protein (eGFP) transcription to produce pEP-IR.^27^ The pEP-IR vector was used in transfections of the hematopoietic progenitor cells CD34^+^, resulting in appreciable amelioration of all transfection parameters relative to the control vector, which carried only the S/MAR element. These observed improvements included an increase in transfection efficiency in CD34^+^ cells from ∼25% to ∼32% relative to the control, a three-fold increase in the rate of vector establishment, a 50% increase in the plasmid copy number per fluorescent cell, an increase in the rate of eGFP mRNA per plasmid copy relative to the control vector (∼3 times), and finally, resulted in long-term transfection (Fig. 3C).^27^ Furthermore, the IR, as part of a pEPI-1-based vector designed to carry an activator of the γ-globin gene, again modulated a positive effect in all the transfection parameters as mentioned above.^28^ Thus, the IR element, a mammalian origin of replication, has an important role to play in the replication of S/MAR-based autonomous vectors.

In light of data presented by Hagedorn *et al.*,^43^ which documented that vector establishment sites are located near origins of replication within the respective nuclear compartment, we consider that the IR may act as a genomic, *cis*-acting origin of replication for the S/MAR element. Furthermore, the IR may favor the formation of an autonomous plasmid replicon element, when it resides in the same episome as the S/MAR element. In support of these considerations are reports on IR association in its natural, genomic position with the MAR element identified in the Locus Control Region of the β-globin gene cluster. This promotes the formation of higher order chromatin structures required for the transcriptional regulation of the globin genes during development.^49^

Considering the overall picture and advances in the development of nonviral, episomal vectors, it is evident that an orchestrated and well-balanced network of interactions exist among the main players that support the episomal status of the vector within the eukaryotic nucleus, namely the compartmentalization of the nuclear matrix and epigenetic factors deriving from the host cell on one hand and the S/MAR, the origin of replication, the insulator sequences, the backbone sequences residing on the vector, on the other hand. Furthermore, new insights provided by the knowledge of the preferred sites of interaction between the plasmid and the nuclear matrix mediated by insulators and the additional strengthening of episomal function of S/MAR-based vectors brought about by the strong mammalian replication IR, present a new perspective for the development of nonviral, episomal vectors.

## Episomal Vectors Devoid of Harmful DNA Sequences

Episomal vectors based on the S/MAR element do not code for any viral protein, which is a significant aspect of their safety profile. Nevertheless, “harmful” DNA sequences exist in a replicating episome, and can reside in the promoter,^50^ transgene,^51^ or backbone.^23^ These sequences are often targets of epigenetic gene silencing,^23,52^ as well as the genes within the vector that are necessary for plasmid propagation in bacteria. The deletion of such sequences generally produces smaller constructs, which may improve the delivery into (and movement within) the recipient cell. The genes for antibiotic resistance and the origin of prokaryotic replication may harbor a number of CpGs, which are involved in heterochromatin expansion leading to transgene silencing and inducing immune responses.^53^ However, such effects may also be derived from other backbone sequences, regardless of the presence of the CpGs.^54^

Within plasmids, CpG sequences are more often present in DNA of bacterial origin than in mammalian DNA. Following gene transfer, these sequences can be recognized by the mammalian immune system through the Toll-like receptor 9. The immune system reacts by releasing proinflammatory cytokines, resulting in inflammation, which can be circumvented by depletion of the CpG motifs from the vector as shown for AAV, for example, followed by improved outcomes in clinical trials for a skeletal muscle disease.^55,56^ As a result of such data, the AAV vectors currently used have a reduced CpG content. Nevertheless, in clinical trials for correction of hemophilia with CpG-reduced AAV8 vectors, the recipients of high vector doses exhibited an increase in AAV capsid-specific CD8^+^ T cell responses following gene transfer.^57^

Most currently, during the application of gene therapy for X-linked myotubular myopathy (MTM), three MTM subjects unfortunately died, two of them from progressive hepatobiliary disease and subsequent sepsis related to systemic inflammation,^58^ and one from gastrointestinal bleeding.^59^ The mechanism by which this toxic effect incurred is unknown, but the focus of these hypotheses is either a pre-existence of antibodies or their rapid accumulation after infusion.

### Vectors depleted of CpG motifs

**Figure 4.**
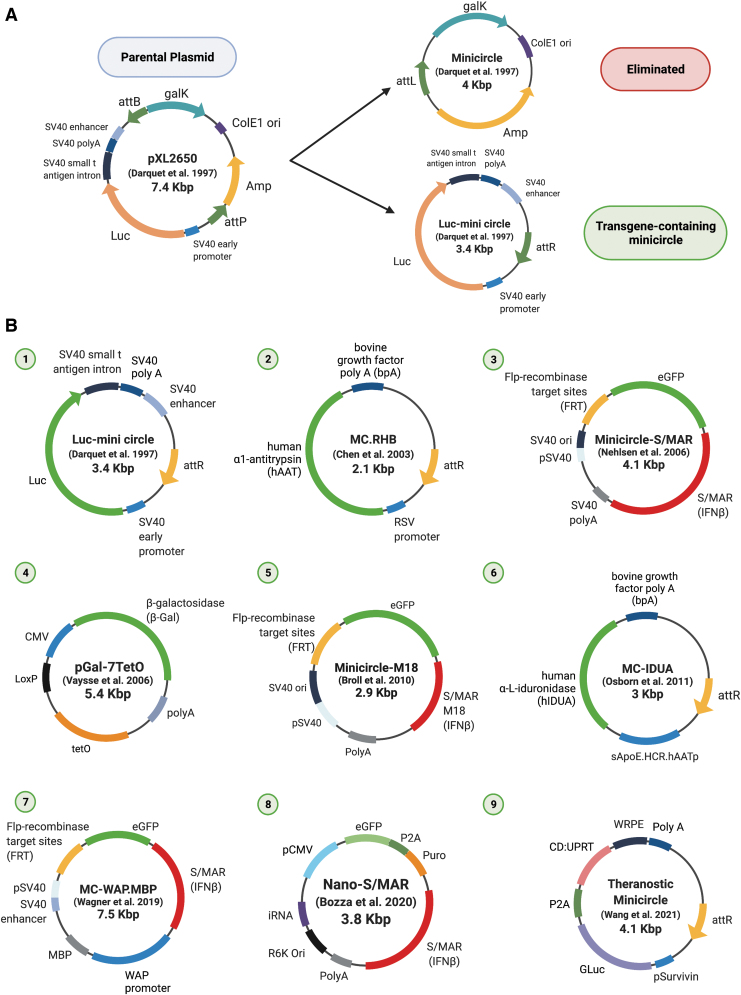
A “minicircle” family of vectors relating to S/MAR episomes. **(A)** An example^68^ of how MC is generated, showing how transgene (Luc) containing MC can be excised from a parental plasmid (pXL2650), with concomitant loss of the nontransgene containing portions of the vector. **(B)** A list of key vectors in the “minicircle” family, and several shown here contain S/MAR elements. Vector (1) in the scheme, produced by *in vivo* recombination in *Escherichia coli* by att site-specific recombination mediated by the phage λ integrase. It is devoid of bacterial antibiotic resistance marker and an origin of replication.^68^ Vector (2) contained the Rous sarcoma virus long terminal repeat promoter and mediated the production of super helical MC DNA through the C31 integrase, a more efficient recombinase relative to others, resulting in transgene expression 45- to 560-fold higher and more persistent compared to conventional plasmids *in vivo.*^69^ Vector (3) is an MC that consists of an active transcription unit and the S/MAR, which replicates stably without integration.^70^ Vector (4) is a nuclear-targeted MC to enhance gene transfer of nonviral vectors *in vitro* and *in vivo.*^71^ Vectors (5) are replicating and contain either a 2 kb (MC) or a 733 bp (M18) portion of S/MAR.^72^ Vector (6) was used in preclinical studies, with the use of a tissue-specific promoter (sApoE.HCR.hAATp) in conjunction with microRNA target sequences, for producing hIDUA, resolving mucopolysaccharidosis type I in mice.^73^ Vector (7) is a self-replicating episomal MC that can persist through stages of serum starvation and nuclear transfer procedures, is stably established in primary bovine cells, and can be developed into MC-containing bovine embryos.^74^ Vector (8) is a nanovector used most recently as a nonintegrating platform for the safe, rapid, and persistent manufacture of recombinant T cells.^75^ Vector (9) is the first activatable cancer theranostic MC system, co-encoding for GLuc and CD:UPRT transgenes on a single surviving promoter-driven construct to create an all-in-one theranostic MC for prostate cancer.^76^ bpA, bovine growth factor polyadenylation signal; CD:UPRT, cytosine deaminase:uracil phosphoribosyltransferase; GLuc, Gaussia luciferase; hAAT, human α1-antitrypsin; hIDUA, human α-l-iduronidase; MBP, myelin basic protein cDNA expression cassette; MC, minicircle; pSurvivin, Survivin promoter/baculoviral inhibitor of apoptosis repeat-containing 5 (BIRC5); RHB, RSV.hAAT.bpA expression cassette; RSV, Rous sarcoma virus long terminal repeat promoter; WAP, whey acidic protein; WRPE, Woodchuck hepatitis virus post-transcriptional regulatory element. Created with Biorender.com Color images are available online.

The pEPI-1 vector contains a total of 305 CpG islands in the backbone, and two transcription units consisting of a first CMV-GFP-S/MAR unit and a second SV40-*neomycin phosphotransferase* (NPT) transcription unit. Reducing the CpG content in the pEPI backbone has resulted in plasmids with more efficient transgene expression, as is the case for the pEPito vector, which carries only 37 CpG sites^23^ (Fig. 4A). pEPito thus has a backbone with reduced CpG islands and without the SV40-NPT transcription unit, and has continuously outperformed pEPI-1 in transient transfection efficiency, gene expression levels modulated within stably selected cells, and colony-forming efficiency both *in vitro* and *in vivo*. For all the pEPito-based vectors examined in human embryonic kidney HEK293 cells, ∼75% transfection efficiency was recorded, regardless of the plasmid size, compared to ∼45–65% with all pEPI-1-based vectors. In the NIH 3T3 murine fibroblast cell line, the transfection efficiencies of pEPito-based vectors were, however, at a much lower level (10–16%), but still higher than those achieved with the pEPI-1 vectors (4–13%).

Work with a set of pEPI-derived vectors inducing transdifferentiation of pancreatic β cells in the rat^60^ showed that a reduction in CpG motifs could promote prolonged transgene expression relative to pEPI or pEPI-CMV within the 28 days tested, and a significant increase in insulin levels, suggesting that it could be an optimal plasmid for hydrodynamic gene delivery of transcription factors for therapeutic applications. Furthermore, in an *in vivo* study in rat corneas,^61^ a pCpG-depleted commercial vector had >100-fold higher transfection efficiency relative to the pEPI-CMV, suggesting therapeutic applications are possible for regenerative conditions in future studies.

An interesting and commercially available new version of a CpG-free vector (pCpGfree-mcs) of relatively small size (3 kb)^62^ and minimal bacterial sequences contains a combination of the mouse CMV enhancer, the human elongation factor 1β core promoter, and a 5′UTR (untranslated region) bearing a synthetic intron. The polyA signal is a CpG-free form of the late SV40 polyA. This vector also contains two S/MARs, one from the 5′ region of the human IFNβ gene and a short MAR β-globin sequence, both naturally CpG free. The S/MARs are placed between the bacterial and mammalian transcription units. Current publications suggest the vector is safe and can promote effective therapeutic gene transfer.^63,64^

Other recent work with CpG-free vectors based on this vector has proposed that the position of S/MAR in the 3′UTR of mRNA may not always be ideal in the case of secreted proteins. When the S/MAR was positioned within the transcriptional unit, a decrease in the expression of secreted proteins (SEAP and Lucia) was observed, possibly by altered nuclear export mechanisms relative to nonsecreted gene products. These observations by Bruter *et al.*^65^ suggested that the requirements of vector CpG-free sequences to optimize gene expression can vary depending on the tissue to be transfected. Interestingly, the same group reported that for mouse liver^65^ or human mesenchymal stem cells,^52^ both the vector and the transgene should be CpG free, whereas in mouse skeletal muscle,^65^ removing the CpG motifs from the vector alone was sufficient to maintaining expression for about 100 days relative to control vector. Noticeably, other vectors with similar dual S/MAR regions (IFNβ S/MAR and an immunoglobulin S/MAR^66^ or short chimeric MARs^67^) promote higher colony formation, transgene expression levels, and maintenance in CHO cells relative to one S/MAR, suggesting that more than one S/MAR may augment the vector's activity and function further.

### Minicircle DNA devoid of antibiotic resistant genes

The designation minicircle (MC) DNA refers to nonviral circular vectors devoid of antibiotic resistance sequences and bacterial origins of replication, and as they cannot replicate, they are not considered plasmids. Their production is based on an *in vivo*, intramolecular recombination of the parental plasmid at *attP* and *attB* sites, driven by the *Escherichia coli* bacteriophage l integrase, and an example is shown in Fig. 4A. This results in an MC, carrying sequences for eukaryotic expression, and a miniplasmid with the undesired backbone sequences, as first reported by Darquet *et al.*^68^

Extensive studies on the MC performance *in vitro* and *in vivo* have revealed its important capacities, such as high stability of gene transfer, substantial reduction of toxicity, enhanced and persistent transgene expression, and minimum side effects.^27^ *In vivo* persistence and high-level expression of MC DNA were demonstrated early on, by transfection into mouse liver, with 45- and 560-fold more serum human factor IX and α1-antitrypsin expression levels, respectively, compared to control plasmids.^69^

MC has been used to induce pluripotent stem cells, and have been used in successful preclinical studies.^27^ Therapeutically, MC that efficiently transfects a number of cell types such as liver and lung epithelia, and hematopoietic stem and progenitor cells, has been used for the development of integration-free (chimeric antigen receptor) CAR-T cells, for generating DNA vaccines. A series of MC has been generated, many of which contain S/MAR, which are included in Fig. 4B. For instance, the MC.RHB.RSV, containing the Rous sarcoma virus long terminal repeat promoter, could mediate the production of super helical MC DNA mediated by C31 integrase, a more efficient recombinase relative to others, resulting in transgene expression 45- to 560-fold higher and more persistent compared to conventional plasmids *in vivo.*^69^

The MC-S/MAR, an MC that consists of an active transcription unit and the S/MAR, replicates stably without integration,^70^ whereas the *pGal-7TetO* (TetR-NLS or TetR-TAT) is a nuclear-targeted MC that enhances gene transfer of nonviral vectors *in vitro* and *in vivo.*^71^ The MC MC/M18 is replicating and contains either a longer (MC) or a shorter (M18) portion of the S/MAR element.^72^ The *MC-IDUA* has been used in preclinical studies, with the use of a tissue-specific promoter (sApoE.HCR.hAATp) in conjunction with microRNA target sequences, for producing human α-l-iduronidase, successfully resolving mucopolysaccharidosis type I in mice.^73^

MC also has been stably transmitted in clonal primary bovine fibroblasts and are maintained through the process of somatic cell nuclear transfer and early embryonic development. These data are highly promising for the use of episomal MC for the generation of transgenic animals.^74^ The *MC-WAP.MBP (MC-S/MAR)*, stably established in primary bovine cells, is a self-replicating episomal MC that can persist through stages of serum starvation and nuclear transfer procedures, and can be developed into MC-containing bovine embryos (Fig. 4).^74^ Finally, most recent advancements are the Nano-S/MAR, a nanovector used most recently as a nonintegrating platform for the safe, rapid, and persistent manufacture of recombinant T cells,^75^ and an activatable theranostic MC, co-encoding for GLuc and CD:UPRT transgenes on a single surviving promoter-driven construct to create an all-in-one theranostic MC for prostate cancer (Fig. 4).^76^

### The pFAR and nano-S/MAR vectors free from antibiotic resistance genes

**Figure 5.**
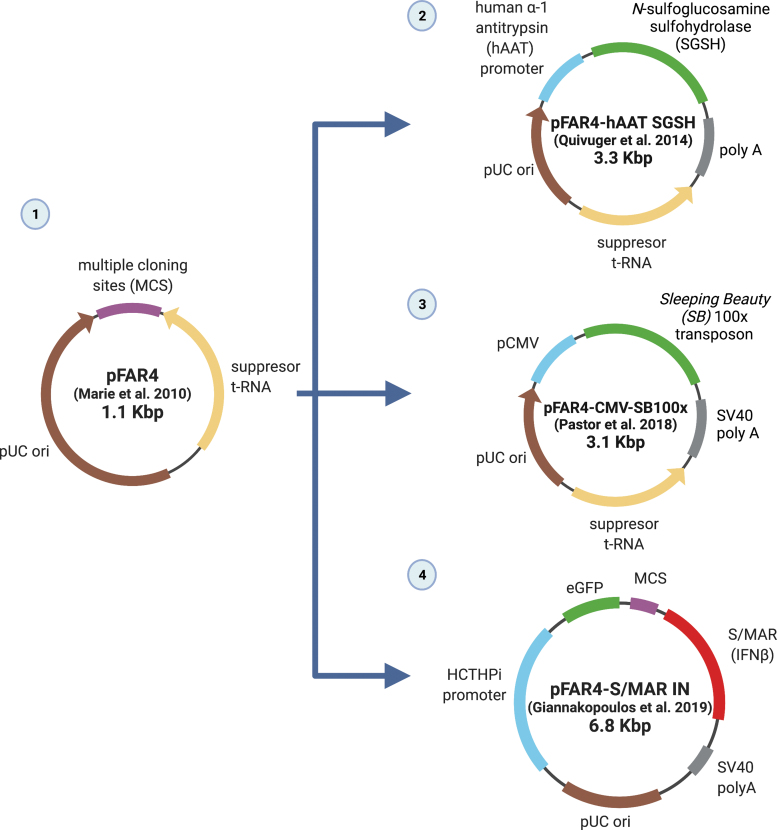
The “pFAR4” family of vectors. (1) The original pFAR4 vector was characterized by its lack of antibiotic resistance markers and its production reliance on the suppression of a chromosomal amber mutation by a plasmid-borne function.^78^ (2) The pFAR4-hAAT-SGSH is a pFAR vector encoding SGSH, which mediated high and prolonged sulfamidase secretion by hepatocytes of MPS-IIIA mice following administration.^81^ (3) The pFAR4-CMV-SB 100 × is a pFAR miniplasmid free from antibiotic resistance genes with the Sleeping Beauty transposon/SB100 × transposase system, and had produced sustainable levels in serum of PEDF following delivery to the liver.^81^ (4) The pFAR4-S/MAR-IN vector mediates efficient episomal gene transfer to hepatic cells for liver-directed gene therapy.^80^ The parental vector was shown in another study to carry reduced heterochromatin marks relative to pKAR4 (Kan^R^ marker containing), which facilitated pFAR4-sustained transgene expression in mouse liver.^79^ PEDF, pigment epithelium-derived factor; SGSH, *N*-sulfoglucosamine sulfohydrolase. Created with Biorender.com Color images are available online.

To minimize the vector size and increase its transfection and expression efficiency, bacterial sequences were removed from the backbone, generating pFAR-4 and nano-S/MAR. These vectors were developed based on an antibiotic-free selection^77^ alternative to MCs, which can often have a laborious production process. The small (1.1 kb) pFAR4 miniplasmid (Fig. 5)^78^ is propagated in *E. coli* through vector engineering, which encodes an amber suppressor codon in the tRNA for histidine. Due to this feature, the vector can rescue normal cell growth of the thymidine auxotroph *E. coli* strain carrying an amber mutation inserted in a thymidylate synthase *thyA* gene.

To assess its potential after electrotransfer in eukaryotic cells, the pFAR4 was combined to the luciferase reporter gene (pFAR4-Luc) and its superior performance relative to controls was documented in muscle, skin, and tumor xenografts.^56^ Importantly, prolonged transgene expression—measured by sulfamidase secretion from the mouse liver (Fig. 3D)—was recorded and related to substantially reduced heterochromatin formation by the pFAR4 miniplasmid at the 5′ end of the sulfamidase gene preventing transgene silencing, compared to the control vector pKAR4 containing the Kanamycin resistance gene, and unchanged methylation status in either of the two plasmids.^79^ Furthermore, the addition of the S/MAR element improved persistence of the pFAR plasmid, while still remaining an intact, free episome, with no detectable vector integration events.^79,80^

These small vectors bear promise for continued translation to larger systems, potentially toward clinical trials, owing to their safety in preclinical studies. Other advancements of these vectors are presented in Fig. 5, and have included the development of the pFAR4-hAAT-SGSH, encoding *N*-sulfoglucosamine sulfohydrolase (SGSH), which mediated high and prolonged sulfamidase secretion by hepatocytes of MPS-IIIA mice following administration.^81^ Also, a pFAR4-CMV-SB 100 × contains the Sleeping Beauty transposon/SB100 × transposase system, and had produced sustainable levels of pigment-epithelium derived factor in serum following delivery to the liver.^81^ A pFAR4-S/MAR-IN vector has been generated, which also mediates efficient episomal gene transfer for liver-directed gene therapy.^80^ In another study, this pFAR4 carried reduced heterochromatin formation, which facilitated sustained transgene expression in mouse liver.^79^

A very recent development in the miniplasmid field is the small “nano”-S/MAR (nS/MAR) vector.^82^ An S/MAR sequence^21^ was introduced into plasmids containing an optimized bacterial backbone (pS/MAR) or minimalistic antibiotic vectors (nS/MAR, Fig. 4). The S/MAR sequence was placed after an expression cassette in which the CMV promoter drives the expression of the reporter gene GFP and the antibiotic selection puromycin divided by a P2A self-cleavage linker sequence. The nS/MAR DNA vectors generated robust transgene expression and a higher efficacy of establishment as episomes in dividing cells relative to pS/MAR, due to the minimal impact on cellular processes and perturbation of the endogenous transcriptome. About twofold more HEK293 colonies were generated using the nS/MAR vector relative to pS/MAR. Transcriptome analysis of the cancer cells revealed that at the molecular level, the presence and the extrachromosomal replication of plasmids carrying bacterial sequences were responsible for altered expression of ∼400 genes, mostly associated with antiviral and inflammation responses. These findings are typical,^83^ since the presence of bacterial sequences in plasmids is responsible for the cell responses against foreign DNA that promote epigenetic silencing.

Most recently, two new vectors were developed from the antibiotic-free nano-S/MAR (SP-nano-S/MAR-B and A).^75^ These two nanovectors showed higher transfection and expression efficiency relative to the parent nano-S/MAR vector and were capable to generate genetically modified human T cells. These vectors should certainly continue to be optimized and applied to a wider variety of cells, as they appear quite promising for therapeutic applications.

## Regulation of Transgene Expression

### The transgene promoters: their effects are cell dependent

It is well established that the transgene promoter type affects the plasmid's gene expression levels. The transgene promoter in an episomal vector can be tissue specific or a generic, constitutive one. Using tissue-specific promoters ensures that the sites of action of the transcription machinery and factors needed to bind on the promoter are fully provided. Generic and hybrid promoters, on the other hand, offer the possibility of studies with an array of different cells, providing valuable information on gene transcription regulation within the constraints of an episome. However, the use of such vectors is restricted, as the results obtained are not uniform across different cell types.

The pEPI vector, a commercial vector conferring robust expression of a transgene in several cell types, carries the promoter CMV-immediate early promoter (IEP) to drive transcription of eGFP and through the S/MAR. In the seminal work by Haase *et al.,*^23^ with pEPI-derivative vectors, the expression levels promoted by the CMV-IEP element were threefold higher in human embryonic kidney HEK293 cells than in murine fibroblast cell line NIH3T3. eGFP expression in this case was cell line dependent and the authors noted that the CMV-IEP promoter seemed to be extraordinarily susceptible to epigenetic silencing within the NIH3T3 cells. The performance of pEPito-based vectors was further improved when the human CMV enhancer/human elongation factor 1 alpha promoter (hCMV/EF-1αP) replaced CMV-IEP, showing that these two promoters have different regulation capacity within the respective chromatin structure of NIH3T3 cells.

It was recently reported^84^ in HEK293 and Chang liver cells that the EF-1αP can induce the highest percentages of highly expressing cells among eight different vectors studied. In addition, the EF-1αP increased the eGFP transgene expression in primary, porcine fetal fibroblast cells by ∼2-fold higher than the CMV promoter in transient and stable cell pools. The authors noted that promoters of endogenous mammalian genes, such as EF-1α, can possibly be more resistant to silencing than viral promoters, which may show variable effects.

In another study, the CMV-IEP promoter within the episomal vector pEPI-eGFP showed variable activity in hematopoietic cells.^27^ This vector had the capacity to transfect and establish long-term transfection culture in human K562 cells and in CD34^+^ human hematopoietic progenitor cells, although only transiently. Specifically, transfected CD34^+^ cells were placed in a semisolid culture for a colony-forming cell (CFC) assay, where every cell produces a colony within 2 weeks. CD34^+^ cells transfected with pEPI-eGFP produced colonies in CFC assay that were all nonfluorescent. When the CMV-IEP promoter was replaced by the hybrid promoter EF-1α/human T cell leukemia virus long terminal repeat (*EF-1/HTLV*) or the *SFFV* promoter, these regulatory elements were able to better sustain eGFP transcription in CD34^+^ and derived cells, with the respective CFC assays showing ∼50% and ∼57% fluorescent colonies, respectively.

When a liver-specific promoter has been used (α1-antitrypsin, AAT-S/MAR) for achieving long-term expression, it could enable expression for up to 6 months in mouse liver,^45^ which is impressive since the control vectors (CMV-S/MAR) could not sustain transgene expression past 1 week due to methylation at several sites within the CMV promoter. The pFAR4 vector also has shown great promise for directing liver-specific transgene expression,^46^ whereby a study used the composite HCRHPi promoter with success in different configurations (relative to the position of the S/MAR). The HCRHPi is a liver-specific human AAT promoter combined with a truncated 1.4-kb human factor ICX intron. Positioning the S/MAR element in an upstream configuration respective to the polyA signal of the eGFP expression cassette showed the highest transfection efficiency and sustained transgene expression for up to 3 months of culture without integration relative to downstream or no S/MAR.

In combination, these data on the widely used promoter CMV compared to other promoters indicate that it is neither possible to extrapolate conclusions from one cell line to the other, nor from cell lines to primary cells and vice versa, and between *in vitro* to *in vivo* conditions. Taken together, these results show the importance of promoter choice as it impacts gene expression levels, persistence, and the effects of triggering epigenetic silencing in various cell types.

### Regulation at the chromatin level/histone dynamics

The regulation of pEPI replication appears to be similar to that of chromosomal DNA; however, the exact mechanism remains incompletely understood. Replication in the cellular genome is tightly regulated so that it occurs once per cell cycle. This regulation is controlled by the assembly of prereplication complexes (pre-RC), consisting of a DNA-bound multisubunit ORC. pEPI-1 replication was studied by Schaarschmidt *et al.*^31^ and findings supported once-per-cell-cycle replication in early S phase. They also showed that the pEPI-1 carries ORC and other components of pre-RC such as Mcm protein binding regions at several regions of the plasmid. Orc1p and Mcm behave similarly when bound to pEPI or to chromosomal locations, from which they dissociate during S phase. In addition, the ORC was found to bind to many DNA sequences *in vivo*, suggesting that the binding of ORC and initiation of replication do not necessarily depend on the underlying DNA sequence, but it is mediated by epigenetic mechanisms.

The episome's chromatin state and its nuclear distribution were investigated using pEPI derivatives in CHO-K1 cells.^85^ This work showed that changes in chromatin state, such as increases in histone acetylation or decreases in DNA methylation modulated by administering either histone deacetylase (HDAC) or DNA methyl transferase (DMT) inhibitors, induce changes in episomal (and the host's) gene activity, as well as influence the episome's nuclear distribution in a dose-dependent manner. HDAC and DMT inhibitors promoted increased numbers of fluorescent cells and also in the average fluorescence intensity observed. Episomes were found to be immobile within the nucleus during a period of tens of minutes, possibly anchored by the S/MAR element. However, episomes apparently could relocate to positions closer to the nuclear center if their gene expression was upregulated by targeting specific promoter domains, just as it has been reported for chromosomal gene activity, under treatment with these HDAC or DMT inhibitors. This report essentially suggested that episomal genes behaved remarkably similar to host genes.

The distribution of histone modifications has been compared for active chromatin in pEPI and pGFP vectors.^86^ While H3K4me3 was detected in all elements within pEPI-eGFP (such as the CMV promoter, the GFP gene, the SV40 promoter, the HSV TK polyA, and the S/MAR), this histone modification was highly enriched at the 3′ S/MAR and lower in the 5′ S/MAR, the SV40 promoter, and the polyA regions. Similarly, another histone modification for active chromatin, H3K4me1, was the most concentrated at the 3′ S/MAR region and least concentrated at the SV40 promoter. Interestingly, the distribution also changed with the cell cycle stage, as the S/MAR region lost most of its active histone modifications (H3K4me3 and H3K4me1) during mitosis.

Furthermore, epigenetic gene silencing within episomes might vary with the cell type harboring the episomes. For example, in a MEL^37^ carrying the pEPI–eGFP driven by the promoter CMV-IEP, a rapid loss in eGFP expression was observed at ∼7 days post-transfection, even though antibiotic resistance (driven by an uncoupled promoter, *i.e.,* the SV40 origin region, which includes the early and late promoters) continued for up to 16 weeks. The silenced expression was reversed through the administration of HDAC inhibitors, suggesting that epigenetic silencing of eGFP expression in MEL cells was regulated by histone deacetylation. However, the human chronic myeloid leukemia blast crisis cell line (K562), carrying the same vector, did not display any loss of eGFP expression and was continuously expressing eGFP up to 16 weeks. Thus, since the constitutive promoter CMV is fully functional *in vivo* in mice using a wide range of plasmids, the results suggested that MEL cell-specific epigenetic properties may underlie the differences seen in promoter strength in these cells.

Evidently, many of the regulatory mechanisms of episome replication are not yet well understood and further studies are needed to identify and better understand the function of components that are either crucial in replication or inhibit the process.

### External control of episomal gene expression

An important parameter of episomal gene transfer for gene therapy is to be reversible, so that it could be terminated if needed, and the best means to this end is an “on/off switching” system. Rupprecht *et al.*^24^ developed the Tet^ON^ system in which the tetracycline response element (TRE) is fused to a minimal CMV promoter encoded on the plasmid pTRE-Tight and the reverse transactivator protein (rtTA) is expressed from a conventional CMV promoter (many tissue- or cell-specific promoters have been used also *in vitro* and *in vivo* in other studies). This episome, or pEPI-Tet^ON^, can be “switched on” in the presence of doxycycline (DOX) as it stimulates the binding of rtTA to TRE that triggers transcription. Removal of DOX “switches off” the activity of the episome, but it is reversible by reinducing DOX, although a decrease in maximum transgene expression is observed with each reinduction. While the removal of DOX does not result in complete silencing of the gene, this system has been used extensively *in vitro* and *in vivo* to demonstrate its applicability in regulating gene expression. Thus, the *tet* system is the most advanced and widely used preclinically, yet it has the potential for immunogenicity against its bacterial components. This system has been tested so far in the context of Ad^87^ and AAV,^88^ and could also be highly useful in episome-based gene regulation.

There are several other inducible systems that might be more suitable for clinical translation, including ecdysone based (RheoSwitch Therapeutic System),^89^ used with an Ad vector to deliver interleukin-12 (IL-12) to glioma preclinically and in a Phase I trial with acceptable tolerability.^90^ Also, there are rapamycin-based dimerizer-inducible switches (ARGENT^91^) used with multiple vectors (AAV,^92^ Ad, Lv, and HSV, reviewed in Naidoo and Young^93^). Other interesting systems include the use of promoters controlled by hypoxia, which are responsive to HIF1α (Ad, reviewed in Naidoo and Young^93^), and reversible RNA-based switches, for example, for controlling expression of a delivered therapeutic through engineered ribozymes that can be regulated by an antisense oligonucleotide (AAV^94^). Overall, the ability to regulate both the timing and specificity of gene expression mediated by episomes will be important to maximizing their utility and conceivably, the inducible systems described above can be adapted to these and related vectors.

## Biomedical Applications of Episomes

### Genetically modified animals

Genetically modified animals are important as models in biotechnology and biomedicine research. A study done by Manzini *et al.*^95^ highlights the success in generating genetically modified pigs using pEPI-eGFP through sperm-mediated gene transfer. About 70% of the fetuses demonstrated presence of the vector in skeletal muscle, heart, liver, kidney, and lung tissues without DNA integration. A more recent study by Wagner *et al.*^74^ has explored a similar idea with MCs, and showed that these remained episomal in bovine fibroblasts for more than 2 months. They also tested these vectors to examine if MC could endure the process of somatic cell nuclear transfer and found that cells outgrown from blastocysts maintained the MC through early embryonic development, supporting the prospect of using cell lines with episomal MC for generating transgenic animals. Despite the relatively more transient nature of transgene maintenance over time in episomal transgenics, important advantages could include lack of integration into the genome and providing the basis for germline gene therapy, which would permit treatment of genetic diseases at the time of conception, as proposed for swine and mouse reproductive biology applications.^95^

### Delivery of RNA interference/short hairpin RNA

RNA interference (RNAi) has great potential for inhibiting gene expression in gene therapy applications. While the use of siRNA can efficiently inhibit gene expression, the effect is only transient in mammalian cells. Taking advantage of the pEPI-1, Jenke *et al.*^96^ introduced a short hairpin RNA (shRNA) expression cassette targeting the *bcr-abl* oncogene in a human, chronic myeloid leukemia blast crisis cell line, K562. Results show that 42 days post-transfection, there was a ∼90% reduction in bcr-abl expression in K562 cells transfected with the episomal shRNA vector. Interestingly, the suppression of *bcr-abl* in these cells was highly specific as it did not affect the bcr protein expression, but rather only the *bcr-abl* fusion mRNA. Long-term effects were also observed with stable maintenance of the episome and gene silencing even after four additional months of cell culture in the absence of selection pressure.

Episomes could also more efficiently sustain expression of shRNA-based elements for antiviral applications. For example, pEPI vectors have been used to target different Hepatitis B virus (HBV) mRNAs through shRNA^97^ in an attempt to prevent the emergence of resistant viral strains. At 4 weeks post-transfection, vectors delivering RNAi suppressed 70–90% of the Hep B surface antigen cellular expression relative to control vectors. A prolonged effect was also observed at 8 months post-transfection in the absence of selective pressure. At 3 months post-transfection, the RNAi vectors also inhibited intracellular HBV DNA replication by 70–90%, as assessed by comparison with pregenomic HBV RNA levels. While these vectors do not seem to have been tested *in vivo* yet, these results showed promising potential for episomes delivering RNAi as an innovative nonviral based treatment.

### pEPI vectors for delivering therapeutic genes

A recent study conducted by De Rocco *et al.*^98^ evaluated the possibility of using episomes in developing therapies for cystic fibrosis (CF) patients. Currently, drug delivery combinations can improve lung function for some patients, yet therapeutic success is still limited. Since CF is a single gene disorder due to mutations in the cystic fibrosis transmembrane conductance regulator (*CFTR*) gene, introducing the wild-type copy of the gene was the first therapeutic approach applied by retroviral gene transfer. In this study, the authors examined S/MAR-based pEPI vectors in bronchial epithelial cells. Vector pBQ for the *CFTR* gene showed some episomal persistence, with ∼25–35% GFP-positive cells at 2 days post-transfection, but it was gradually declining to <1% after only 2 weeks. The vector pBQ-S/MAR was constructed and used to transfect bronchial epithelial cells. The *E. coli* rescue assay showed that the vector pBQ-S/MAR was still present on day 14 post-transfection relative to pBQ, which was lost by day 10. CFTR mRNA and CFTR protein expression were also stably detected. These data suggest that the S/MAR element can improve the vector persistence and long-term CFTR production in bronchial epithelial cells. However, the vector does not yet appear to be optimized for maintenance past 2 weeks.

Additional advances have included contributions in ocular gene therapy, including the long-term genetic correction of disease in a mouse model of a debilitating ocular disease called Leber's congenital amaurosis. In this study, pEPI-based S/MAR vectors were used to phenotypically rescue ocular pathology and contained a vitelliform macular dystrophy 2 promoter driving retinal pigment epithelium-specific expression of RPE65 in *rpe65*^−/−^ mice.^17^ The vector was formulated and delivered by CK_30_PEG10k^99^ (polylysine-PEG) nanoparticles into the eye, with gene expression lasting for up to 2 years.

### Tumor-specific episomes

Typically, tumor-specific expression *in vivo* can be achieved by transfer vectors that deliver the gene of interest by using tissue-specific expression cassettes, as previously discussed. The modification of a constitutive promoter in pEPI with tumor-specific promoters can generate a vector that episomally expresses the transgene in tumor cells.^100^ For example, pEPito derivatives have been compared with constitutive CMV or EF-1α promoters and with alpha fetoprotein (AFP) promoter in liver carcinoma. The study showed that the AFP plasmid containing an hCMV enhancer (hCMV/AFP) boosted the expression activity by ∼15- to 20-fold in hepatoma cell lines. Although a CMV-driven plasmid had considerable luciferase activity in lung tissues, it was expressed ∼8 times lower in tumors, whereas the hCMV/AFP plasmid promoted luciferase activity in the lungs and levels of expression in tumors were maintained. Taken together, this shows the functionality in the hCMV/AFP element in promoting specificity and sensitivity for hepatocellular carcinoma both *in vitro* and *in vivo.*

Finally, a significant advance has been the reported use of the nano-S/MAR miniplasmid for genetically modifying primary pancreatic cancer cells^82^ with a particular focus on vector-mediated toxicity and an analysis of the molecular integrity of engineered cells. The nS/MAR vector was capable of providing sustained genetic supplementation of the tumor suppressor SMAD4 in pancreatic cancer models. The expression of a key tumor suppressor gene was restored, and the engineered cells retained stable expression of the transgene *in vivo*. The histopathological analysis of tumors showed that the modification of the cells with this vector technology had a minimal impact on the cells' behavior, as they formed tumors that displayed a highly differentiated pancreatic adenocarcinoma morphology similar to tumors formed from the unmodified parental cell line. The metastasis in the liver and in the lungs of mice injected with parental cells also confirmed that the vector did not alter cellular behavior, and tumors retained their aggressive metastatic potential.

The nS/MAR system can be considered a potent tool for persistently modifying cells, providing sustained transgene expression, while avoiding risks of insertional mutagenesis and other vector-mediated toxicity. nS/MAR vectors show promise to be a valuable genetic tool for generating persistently modified isogenic cells, enabling transgene expression with minimal vector-mediated impact in cultured cell lines or primary and patient-derived cells.

### The clinical translatability of episomes

To our knowledge, there has been no therapeutic application of episomes in clinical trials, yet recent advancements made in these DNA vectors should make it desirable and feasible to translate these vectors. The existing state-of-the-art for clinical trials is to use conventional (highly transient) plasmids. For example, there are ∼280 clinical trials (current or recently completed) in the *U.S. National Library of Medicine database*^101^ for the search terms “pDNA” or “plasmid.” The applications range from musculoskeletal or ischemic limb repair to vaccines and cancer therapeutics.

A gene commonly delivered using plasmids is the human interleukin-12 (hIL-12). Some highlights include recently completed Phase I trials examining the safety and efficacy of phIL-12 plasmids formulated with PEG-PEI-cholesterol (PPC) by Celsion/EGEN, and other phIL-12/PPC Phase I and II trials by the Gynecologic Oncology Group/NCI and Celsion, administered intraperitoneally for treating ovarian neoplasms. Oncosec Medical Incorporated is examining delivery of another hIL-12 plasmid, tavokinogene telseplasmid, by electroporation intratumorally in several current or completed Phase II trials for advanced melanoma, triple negative breast cancer, metastatic head and neck squamous cell carcinoma, and Merkel cell carcinoma.

Another plasmid has been tested by Synergene Therapeutics that is using plasmid SGT-53 in several Phase I and II trials for treating metastatic pancreatic cancer and childhood CNS tumors through intravenous delivery. SGT-53 is a complex composed of a plasmid carrying the wild-type p53 cDNA and encapsulated with a tumor-targeted (anti-transferrin receptor single-chain antibody fragment) cationic liposome. University Hospital-Toulouse, in collaboration with InvivoGen Therapeutics, initiated a phase II trial to determine the clinical activity of CYL-02, a nonviral gene therapy product that sensitizes pancreatic cancer cells to chemotherapy. This plasmid delivers a mouse somatostatin receptor subtype 2 and a fusion of human deoxycytidine kinase:uridine monophosphate kinase (DCK:UMK) complexed with polyethyleneimine (PEI). Following intratumoral administration, this plasmid expresses the DCK:UMK fusion protein, which converts gemcitabine into its antitumor phosphorylated metabolite for treating pancreatic adenocarcinoma.

Another database researched included the Wiley's *Journal of Gene Medicine,*^102^ which lists ∼480 gene therapy clinical trials worldwide using plasmids, and these comprise ∼15% of all vectors used (viral and nonviral). The most common genes delivered are IL-12, GM-CSF, and HSV-TK, yet none actively utilizes episomal vectors as of yet.

The approved products also appear to be restricted to traditional plasmids thus far. For example, *Neovasculgen*, a plasmid-based product carrying the vascular endothelial growth factor gene, is injected directly into target ischemic tissue to stimulate blood vessel growth, and has been available since 2012 for the treatment of atherosclerotic peripheral arterial disease (PAD), including critical limb ischemia. Intramuscular injection of a single dose of this product, costing less than $50, stimulates angiogenesis and blood supply to decrease the risk of amputation and death in patients suffering from PAD. A recent 5-year follow-up study post-therapy revealed a significant increase in pain-free walking distance by PAD patients and confirmed the therapeutic efficacy of this drug.^103^

We believe episome vectors described in this review could be translated similarly to regular plasmids, in particular when used in combination with gene delivery systems that can promote efficient episome entry or uptake into cells. More numerous preclinical studies with episomes eventually should translate to an increase in the pipeline of episomes to be potentially utilized in the clinic.

We can envisage that current efforts would be expected in the coming decade to continue to produce episomal vectors with reduced bacterial sequences, CpG content, reduced size, even more favorable cost-effectiveness, and ease of production, and to continue to improve purity, as well as to increase the range of gene products to also include RNAi delivery. Eventually, changes would enable routine drug-activatable and tunable transcription control through emerging systems such as Rheoswitch or others that might evade immunogenicity challenges.

## Conclusions and Future Directions

Genetic therapies are considered very complex therapeutic systems, although many have already demonstrated efficacy in preclinical and clinical studies. Despite many advances, the vast majority of clinical trials still use viral vectors and several challenges remain, including addressing genotoxicity from integrating vectors, improving gene transfer, achieving ideal expression levels, and avoiding immune responses to viral vector sequences. An important advance has been the use of chromosomal elements that derive from the human genome and do not cause apparent toxicity. Thus, the inclusion of the specific chromosomal element S/MAR, in episomal vectors, had a decisive effect in addressing the problem for plasmid loss and nuclear retention of the vector, leading to long-term transfection, while the addition of the IR element had an extended effect in the generation of stable transfection in hematopoietic primary cell cultures.

New insights on the S/MAR based episomes are also provided by current knowledge of the preferred sites of plasmid attachment onto the nuclear matrix, mediated by chromosomal elements (insulators) within the vector, which promote plasmid establishment onto the nuclear compartment following plasmid arrival at the nucleus. Evidently, the search for more sequences of human origin and more effective insulators may be of crucial importance for further enhancing the efficiency of episome maintenance and promoting sustained, high-level gene expression in cells for gene therapy applications.

Furthermore, most of the advances made to nonviral vector engineering have involved reducing vector length and removing problematic DNA sequences. Each of these advances, however, has brought new problems for the research community to solve—issues with purity and scale. The possibility of utilizing nonviral vectors that are maintained long term as episomes represents undoubtedly one of the most interesting and intriguing fields of gene therapy research.

New innovations will certainly continue to arise to optimize nonviral vectors, enhancing their potential for scale-up and suitability for clinical use. Compared to lentivirus systems, MC vectors support short-term transgene expression, but prevail in biosafety, low and minimum side effects. As long-term persistence and transgene expression are documented properties of a number of current episomal vectors, the next trend that will follow is an increase in preclinical and clinical trials.
